# Tracking malaria health disbursements by source in Zambia, 2009–2018: an economic modelling study

**DOI:** 10.1186/s12962-022-00371-2

**Published:** 2022-07-21

**Authors:** Michael Mtalimanja, Kassim Said Abasse, Muhammad Abbas, James Lamon Mtalimanja, Xu Zhengyuan, DuWenwen, Andre Cote, Wei Xu

**Affiliations:** 1 School of International Pharmaceutical Business, China Pharmaceutical University, Nanjing, 211198 Jiangsu China; 2 Centre de Recherche en Gestion Des Services de Sante, Faculté Des Sciences de L’administration (FSA), Université Laval (UL), Centre Hospitalière Universitaire (CHU) de Québec UL-IUCPQ-UL, Québec, QC Canada; 3 Riphah Institute of Pharmaceutical Sciences, Riphah International University, Islamabad, Pakistan; 4 Department of Monitoring and Evaluation, Ministry of Health, P.O Box, 30205 Lusaka, Zambia

**Keywords:** Malaria interventions, Disease Burden, Malaria funding, Zambia

## Abstract

**Background:**

Zambia has made profound strides in reducing both the incidence and prevalence of malaria followed by reducing malaria related deaths between 2009 and 2018. The number of partners providing malaria funding has significantly increased in the same period. The increasing number of partners and the subsequent reduction of the number of reported malaria cases in the Ministry of Health main data repository Health Management Information System (HMIS) stimulated this research. The study aimed at (1) identifying major sources of malaria funding in Zambia; (2) describe malaria funding per targeted interventions and (3) relating malaria funding with malaria disease burden.

**Methods:**

Data was collected using extensive literature review of institutional strategic document between the year 2009 to 2018, assuming one-year time lag between investment and the health outcome across all interventions. The National’s Health Management Information System (HMIS) provided information on annual malaria admission cases and outpatient clinic record. The statistical package for social sciences (SPSS) alongside Microsoft excel was used to analyze data in the year 2019.

**Results:**

The investigation observed that about 30% of the funding came from PMI/USAID, 26% from the global funds, the government of Zambia contributed 17% and other partners sharing the remaining 27%. Multivariate regression analysis suggests a positive correlation between reducing reported malaria disease burden in HMIS 2009–2018 and concurrent increasing program/intervention funding towards ITNs, IRS, MDA, and Case Management with r^2^ = 77% (r^2^ > 0.77; 95% CI: 0.72–0.81). Furthermore, IRS showed a p-value 0.018 while ITNs, Case Management and MDA having 0.029, 0.030 and 0.040 respectively.

**Conclusion:**

Our findings highlight annual funding towards specific malaria intervention reduced the number of malaria admission cases.

**Supplementary Information:**

The online version contains supplementary material available at 10.1186/s12962-022-00371-2.

## Background

Malaria infections are caused by a *plasmodium* parasite which spreads to people through the bites of infected female *Anopheles* mosquitoes. In 2017, an estimated 229 million cases of malaria were recorded worldwide and 409,000 people died, mostly children in the African region [1]. Malaria occurs in more than 100 countries and territories. About half of the world's population is at risk. Large areas of Africa and South Asia and parts of Central and South America, the Caribbean, Southeast Asia, the Middle East, and Oceania are considered areas where malaria transmission occurs. In the global community, about 111 countries globally have eliminated malaria and another 35 countries, are making progress toward elimination of the disease [2]. The African region accounts for most global cases of malaria (88%), followed by the Southeast Asia region (10%) and the Eastern Mediterranean region (2%).

In Zambia *plasmodium falciparum* malaria is endemic throughout the country, with the main transmission season being between November and March every-year. The Malaria Indicator Survey (2008) indicates that the country’s average parasite rate was found to be 10%, with some parts of the country reporting less than 1%, while others still have high parasite prevalence rates of up to 20%-30% [3, 4].

Malaria has for a long time remained the leading cause of morbidity and mortality in Zambia with recent statistics suggesting malaria still being the leading cause of morbidity and the second leading cause of mortality, surpassed only by HIV and AIDS. Also, malaria accounts for up to 40% of all infant mortality and 20% of all maternal mortality in Zambia, and represents a major socio-economic burden on the country, particularly on the communities living in malaria endemic areas [5, 6].

Zambia has shown strong growth in the last decade, reaching lower-middle-income status. Nevertheless, the health sector continues to be dependent on external resources, which has accounted for over 60% percent of health expenditure in recent years. Out-of-pocket health expenditure contributes 12.8% to total health expenditure. Private medical schemes and insurance account for about 5% [7].

Malaria remains a major public health problem in Zambia, despite significant progress made in fighting the disease in the last decade. Malaria prevalence varies across all provinces and districts with 18 million people at risk, including the most vulnerable groups, such as pregnant women and children. The country’s last two iterations of the National Malaria Strategic Plan aimed to reduce transmission and in the current NMSP (2017–2021) the government of Zambia through the ministry of health and the national malaria elimination program (NMEP) adopted an ambitious agenda to eliminate malaria by use of scientific proven interventions for prevention, control, curative, and inclusion of new tools of innovations in strengthening of routine surveillance at all levels. The efforts towards nationwide malaria elimination with regard to malaria case management, emphasizes the need to have diagnostic and curative services as close to home as possible, utilizing community health workers as extensions for the health facility within the community [8, 9, 10]. Despite a better understanding of pathophysiology and management of malaria, childhood mortality remains unacceptably high [11].

Over the past 10 years, Zambia has significantly intensified efforts against malaria by initiating and scaling up the implementation of internationally accepted strategies and best practices for prevention, treatment, and care for malaria [12, 13]. These include: vector control, through indoor residual spraying (IRS) and the promotion of ownership and correct use of insecticide-treated bed nets (ITNs); intermittent preventive treatment in pregnancy (IPTp); prompt and effective malaria case management; Coartem (artemether/lumefantriine) use; and the introduction and scaling up of Rapid Diagnostic Tests (RDTs) in health facilities that do not have microscopy services [14]. On the other hand, if no new control measures are developed, the malaria death toll is expected to raise as actual figures could be potentially higher as a result of under-reporting and challenges in diagnosis [15].

It was observed that the increasing malaria disease burden can only be contained through harnessing, harmonization and coordination of all the available resources, to maximize the benefits from synergies. In this respect, the national malaria control program (NMCP) has successfully established strong partnerships with the communities, other government line ministries and departments, the faith-based health sector under the coordination of the private sector, civil society, and the global community. Strong, effective, and coordinated partnerships have been established with the global community, through the RBM Partnerships, leading to significant technical, financial, and logistical support. Also, reference documents such as the world malaria report present a scope on source of funding partners and reflects their mandate and area of support. However, provided funding is sustained there is need to harness the fragmented information into one document thereby providing an opportunity to gauge and categories related interventions. In addition, the main data repository for ministry of health (DHIS) only present data in its raw form without linking the disease burden to available funds and interventions. The researcher believes that malaria disease burden, (trends and prevalence) cannot be understood fully unless there is an attempt to discuss the disease burden in light of the country’s funding profile and specific malaria interventions. Therefore, study aims at (1) identifying major sources of malaria funding in Zambia; (2) describing malaria funding per targeted interventions and (3) relating malaria funding with malaria disease burden between 2009 and 2018.

## Methods

### Study design

A retrospective cross-sectional study focused on institutional documents of the ministry of health main data repository. We performed a time series regression analysis with delta of one year factoring in data collected at time point 2009 to 2018 to capture any change that could have been repeated at each successive equally spaced time points to follow a sequence and an average of all time point taken to enable investigation of the pattern of change over time. Statistical package for social sciences (SPSS) alongside STATA version 16, were used to describe data and explain the relationship between the dependent variable malaria disease incidence by adopting and including seven (7) independent variables; insecticide treated nets (ITNs), case management (CM), indoor residual spray (IRS), mass drug administration (MDA), monitoring and evaluation (M&E), entomological studies (ES), information education and communication (IEC).Also stationarity test and variance inflation factor were employed on variables to investigate predictability and collinearity among variables respectively.). Thus, we adopted and included all the seven predictors related to the dependent variable into the model taking a form of standard multiple-linear equitation of the form Y = a + Bx_1_ + BX_2_ + BX_3_**.**

Statistical analysis used descriptive statistics that were quantitatively summarized from a collection of information from documents which included annual financial reports, and partner country operational plans which provide data on actual annual disbursement while other documents like medium term expenditure frameworks, strategic plans, evaluation of country programs, to mention but a few provide data on the budgets estimates and their assumption. Interestingly, all contributions received by the ministry of health from varies partners are expressed as annual disbursement. Also, administrative data such as malaria incidence and admissions were extracted from the District Health Management Information System accessed on (http://www.dhis2.org.zm/hmis).

Hypothesis testing was employed using the ANOVA single factor analysis of variance on malaria.

In addition, prior to data collection, ethical clearance was sought and granted in China from the institutional Review Board of China pharmaceutical University and approval from relevant authorities including Ministry of Health Public Health and Research Unit Zambia.

## Results

Additional file 1 Shows funding partners and reflects their mandate and area of support in the period under review. Contributors to malaria funding for malaria prevention, treatment and control in Zambia included Presidential Malaria Initiative (PMI-USAID), The Global Fund (GF), World Bank (WB), World Health Organization (WHO), United Nations International Children's Emergency Fund (UNICEF), Program for Appropriate Technology in Health (PATH), Malaria Control and Elimination Partnership in Africa (MACEPA) and Department for International Development (DfID) United Kingdom among others.

Disbursements directed to specific interventions in the study period showed a huge and continuous fluctuation in the annual funding disbursement from government and other stakeholders. According to the proportion of funds towards interventions, about 30% (95% CI: 24.43–33.62) of the funding came from PMI/USIAD, 26% (95% CI: 24.72–28.53) from the global funds. The government contributed 17% (95% CI: 16.03–18.46) with other partners sharing the remaining percentage.

Our findings indicated insecticide treated nets (ITNs) receiving a substantial amount of funding in the past 10 years representing about 34.7% of the total funding followed by IRS with 26.9%, clinical case management 19.0%, monitoring and evaluation 11.2% and information communication education 7.8%. While Support towards provision of mass drug administration and entomological surveillance were at 0.4% and 0.08% respectively Additional file 2. Additional file 3 Shows distribution of malaria intervention summarized as simple counts of proven support provided to ministry of health between the years 2009 and 2018. The results clearly indicates 26 institutions representing 24.1% of all institutions provided support towards Information Education and Communication (IEC), 23 institutions representing 21.3% support towards Indoor Residue Spray (IRS), 22 institutions representing (20.4%) support towards Case Management, 21 institutions representing 19.4% support towards provision of Insecticide Treated Nets (ITNs), 8 institutions representing 7.4% support towards M&E, 5 institutions representing 4.6% support towards Mass Drug Administration (MDA) and only 3 institutions representing 2.8% support towards entomological studies (ES).

Table 1 The District Health Information Management System (DHIS) indicated a systematic decline in number for patients admitted with malaria. Trend analysis of malaria admission showed over 60% (95% CI: 56.42–62.32) reduction from 176,664 to 68,898 admission cases denoting on average a reduction of 140,533 cases between the year 2009 and 2018. Data also suggest variation in annual malaria admission.

**Table 1:** Annual malaria admissions (2009–2018)

Provinces	2009	2010	2011	2012	2013	2014	2015	2016	2017	2018
Central Province	9201	6979	7295	7419	7649	10,135	5593	5730	3735	3061
Copperbelt Province	29,396	31,552	28,597	27,726	23,723	21,265	18,548	15,671	11,009	8675
Eastern Province	52,538	61,271	43,149	27,958	16,447	14,269	11,784	9,068	9977	12,158
Lusaka Province	9727	11,665	8083	4578	5699	4886	2150	3282	2574	1817
Luapula Province	23,754	24,269	21,290	24,415	27,532	25,415	18,359	16,306	11,287	13,456
Muchinga Province	10,506	25,125	22,448	17,496	19,320	15,238	12,507	11,884	8486	7726
Northern Province	8560	14,876	20,836	18,467	20,952	22,635	12,821	10,969	10,910	7813
N-Western Province	10,375	12,401	17,704	18,303	26,144	21,504	15,529	14,584	9717	9479
Southern Province	15,794	16,491	8954	5860	4563	5,299	2130	1598	1038	1083
Western Province	6813	8676	10,218	13,857	11,535	8,903	8206	7131	6110	3630
Total	176,664	213,305	188,574	166,079	163,564	149,549	107,627	96,223	74,843	68,898

Table 2 Shows a notable difference in terms of amount of funds received per intervention, with ITNs, IRS, Case management, Monitoring & evaluation, and IEC receiving comparably larger allocation of funds. While mass drug administration and entomological surveillance showed missing data in the first 6 consecutive years of the review period. The average annual disbursement for all interventions from the year 2009 to 2018 was K32, 739,863 equivalent to USD $ 2,751,248. Furthermore, the highest annual average disbursement was towards ITNs at $5,923,341 while the least annual average was entomological surveillance at $807.

**Table 2:** Annual disbursement per intervention (ZMK)

	2009	2010	2011	2012	2013	2014	2015	2016	2017	2018
ITNs	63,522,011	45,654,132	70,876,431	67,371,945	71,049,155	70,726,365	68,403,575	72,080,785	85,757,995	89,435,205
IRS	45,190,100	41,309,645	56,171,615	55,765,409	61,256,167	65,914,956	50,573,746	45,232,536	59,891,325	64,550,115
Case management	26,858,189	36,965,158	41,466,799	44,158,873	51,463,178	61,103,548	32,743,917	18,384,287	34,024,656	39,665,025
Monitoring and evaluation	8,526,278	32,620,671	26,761,983	32,552,338	41,670,190	56,292,139	14,914,088	(8,463,963)	8,157,987	14,779,936
IEC	17,615,142	16,987,632	19,875,342	14,091,817	15,221,917	14,453,691	13,685,464	12,917,238	17,149,011	16,380,785
MDA	–	–	–	–	–	–	2,000,000	2,000,000	2,000,000	2,000,000
Entomological studies	–	–	–	–	–	–	24,000	24,000	24,000	24,000

USD$ to ZMK as at December 31st 2018,$1 = K11.9

Country currency Zambian kwacha (ZMK)

Table 3 The single factor analysis of variance tests using the following null hypothesis: the number of admissions is equal and the alternative hypothesis: the number admissions is not equal, was performed to test equity of variances of reported malaria admissions by province. Results showed *p*-value < 0.00001 and significant at *p* < 0.05. Thus, we reject the null hypothesis indicating that there is sufficient evidence to suggest that reported malaria cases by provinces are statistically different.

**Table 3:** Single factor analysis of variance (malaria admission)

Anova: single factor				
Summary				
Groups	Count	Sum	Average	Variance
Central Province	10	66,797	6679.7	4,899,936.456
Copperbelt Province	10	216,162	21,616.2	63,846,131.73
Eastern Province	10	258,619	25,861.9	379,115,675.2
Lusaka Province	10	54,461	5446.1	11,339,140.1
Luapula Province	10	206,083	20,608.3	30,100,553.79
Muchinga Province	10	150,736	15,073.6	34,927,448.04
Northern Province	10	148,839	14,883.9	30,058,974.32
N-Western Province	10	155,740	15,574	29,823,030
Southern Province	10	62,810	6281	33,294,314.44
Western Province	10	85,079	8507.9	8,365,391.656

ANOVA						
Source of variation	SS	df	MS	F	*p*-value	F crit
Between groups	4,632,659,295	9	514,739,921.7	8.225696848	< 0.00001	1.985594
Within groups	5,631,935,362	90	62,577,059.58			
Total	10,264,594,657	99				

*p*-value from F-ratio calculator (ANOVA)

Table 4 Predictor variables explaining the relationship between funding and malaria incidence using the 95% confidence interval in the overall regression predictive model found a strong association between increasing funding towards selected key interventions and reducing occurrence of new cases of malaria.

**Table 4:** Regression analysis for malaria admission and funding in the year 2009 to 2018

Regression statistics	
Multiple R	0.881493845
R Square	0.777031399
Adjusted R Square	0.331094197
Standard Error	5.422704859
Observations	7

ANOVA					
	df	SS	MS	F	Significance F
Regression	4	204.9542	51.23855	1.742468	0.039622
Residual	2	58.81146	29.40573		
Total	6	263.7656			

	Coefficients	Standard error	t Stat	*P*-value	Lower 95%	Upper 95%
Intercept	30.12836375	57.64544	0.52265	0.01653	− 217.899945	278.1567
Insecticide treated nets	− 2.02612E−07	1.32E−06	− 0.1536	**0.02892**	− 5.8782E−06	5.47E−06
Case management	− 2.58188E−06	9.36E-06	0.810269	**0.03028**	− 3.2679E−05	4.78E−05
Indoor residual spray	− 3.52625E−06	1.73E-06	− 2.04049	**0.01781**	− 4.0862E−05	3.91E−06
Mass drug administration	− 1.37886E−08	6.58E-07	0.020958	**0.04183**	− 2.8107E−06	2.84E−06
monitoring & evaluation	−3.77532E−09	5.98E−03	− 2.8287	0.17882	− 1.8634E−08	2.76E−08
Entomological	2.12326E−07	4.26E−05	0.586358	0.05739	−1.9876E−07	1.76E−05
IEC	− 3.65433E−08	2.35E−05	− 1.937635	0.07653	− 2.5642E−09	3.12E−08

Boldface indicates Statistical significance (P < 0.05). Estimates are expressed in OR with 95% CI.

Predictor: US$ 1.0

Health outcome: number per case of malaria admission

## Discussion

Ministry of health has benefited from various malaria program interventions funds from more than 47 known partners. Local institutions and companies are also recorded to provide support mostly in interventions related to system straightening, program management and public health awareness. Thus, the study highlights the ministry of health having a long-standing, well established partnerships with a range of multilateral, governmental, nongovernmental, and private organizations providing support towards prevention, treatment and control of malaria in Zambia. It is worth noting that, through the district health management information system of the ministry of health which is mandated to collect routine data on service coverage and disease burden, analysis of malaria data indicated that there is a general decline in the number of reported malaria cases in the country [16]. Trend of malaria admissions falling over the years depicting over 60% (95% CI: 58.6–63.4) reduction between 2009 and 2018. However, the declining malaria disease burden is associated with geographical location [17]. Our research outcomes on single factor analysis of variance established a significant geographical variation in the number of reported malaria cases countywide with Eastern province recording highest number of patients admitted, followed by Luapula, North Western, Muchinga and Northern. While Southern and Lusaka reported lowest number of cases and a similar decline in annual malaria reported cases. On the other hand, Luapula province reported highest number of malaria deaths per annum. Copperbelt, Northern and Eastern provinces also reported high numbers of malaria related deaths. Although the number of reported malaria hospital admissions are seemingly high, the trend analysis showed declining malaria admission across all ten (10) provinces.

Furthermore, research results on insecticide treated nets showed a significant predictor value of 2.02612E−7 third after case management and indoor residual spraying in reducing the malaria burden provided one unit of addition investment towards funding. Interestingly, the use of ITNs in the fight against malaria in our study agrees with what was documented by Lengeler (2004) that in areas of stable malaria transmission, provisions of ITNs have potential to reduced parasite prevalence by 13%, uncomplicated malaria episodes by 50%, and severe malaria by 45% compared to equivalent populations with no nets [18, 19, 20]. Also, a study conducted in Zambia to determine ITNs integrity and insecticide content highlighted more availability of ITNs in households compared to previous years due to increases in funding for malaria control and continuous distribution of ITNs achieved through channels such as Antenatal Care, Expanded Program for Immunization and selected primary schools [21].

Analysis on indoor residual spraying was found to be significant in reducing the malaria disease burden with a 3.52625E-6 predictor value denoting greater change in health outcome per additional investment compared to other interventions in the study. Also, showed a consistent association between implementation of IRS and confirmed malaria case incidence, and a stronger association in reducing malaria disease burden. In Zambia IRS activities are conducted annually and these activities routinely include district-level planning and budgeting for targeted areas, assessment of spray structures, training of spray teams, supervision and monitoring of spray activities [22]

According to MOH (2011), Zambia IRS operations expanded from 5 districts in 2003, to 54 districts by 2014, with support from various partners including US President’s Malaria Initiative (PMI). The 2011–2015 National Malaria Strategic Plan recommended IRS in high-risk areas (a minimum of 85% of all targeted structures) with focalized IRS mounted in response to malaria surveillance data [23, 24].

In rural areas access to health care still remains a significant obstacle due to long distances to health facilities and challenges with transport among others. Consequently, many rural patients with malaria do not present to a health facility in time for treatment [25]. Owing to this, community health workers accustomed to high malaria incidence presumptively treat patients for malaria based on their clinical symptoms and this could potentially reduce the number of confirmed malaria cases recorded at health facilities as these interventions are not captured on time. In addition, clinicians had for a long time practiced a common non-evidence based “fever equals malaria” and treated patients as such without laboratory test /RDT results [26]. Owing to the interventions mentioned, our research findings showed case management to be significant in reducing malaria disease burden from the perspective of routine malaria cases recorded in the HMIS-based clinical records.

Mass drug administration is also a well know malaria prevention and control intervention worldwide. A community randomized step-wedged control trail was conducted in Southern Zambia to access effectiveness of population-wide malaria testing and treatment with rapid diagnostic tests and Artemether-Lumefantrine showed a strong inverse relationship. Moreover, A clear relationship between provision of MDA and reducing HMIS reported malaria burden was noticed in the lower transmission strata, malaria parasite prevalence declined from 7.7% at baseline to 0.5% after the first two MDA rounds, an 87% larger decline than seen in the control group [27]. Similarly, our results showed MDA being significant in reducing disease burden though with the least predictor value of 1.37886E-8 when compared with other interventions analyzed and to the previous studies indicating mass drug administration having a strong predictor power to prevent the spread of the disease. The lower predictor power can be attributed to limited data in the years reported in the study.

However, data for information education communication and M&E were not significant in reducing disease burden and showed a week positive relationship between the intervention and annual malaria reported cases. Thus, this would imply that the more the community is informed or educated about malaria, the more likely they will visit the hospital to seek medical services and as such the number of reported cases recorded is expected to increase. In addition, M&E would suggest the number of reported malaria cases recorded increase as more activities to monitor collection of such data are employed.

Malaria transmission is driven by a complex interaction of the vector, parasite, human host, and the environment, and governed by different ecological and social determinants. Human population increase, developmental activities and associated ecological transformations have a significant impact on malaria epidemiology and have invariably exacerbated the situation. Malaria transmission depends markedly on local environmental conditions and other compounding factors, that is, presence of drug-resistant parasites and insecticide resistant vectors, environmental changes, and economy, poverty levels, climatic changes, natural disasters and political instability, adaptability of malaria vectors to changing environments and limited investment in research, and optimization of malaria vector control programs [28]. Thus, entomological surveillance is also important in the estimation of the expected impact of the various control measures on reducing disease burden though our research outcomes were not significant owing to missing data and also having the least percentage of 2.8% representing the number of institutions supporting the intervention. Data is suggestive of need to sustained investment in entomological surveillance to have adequate samples to measure mean mosquito densities.

In the period reported approximately $ 2.8 million from the selected key interventions was invested into different malaria prevention and curative and diagnostic programs each year. Though the finances ploughed into the key interventions are believed to be potentially higher than the estimation captured in this study, as donations and actual budget allocations from the government of Zambia through the ministry of health would increase the financial investment towards reducing malaria burden if incorporated.

Our findings are suggestive of the pronounced decline in malaria disease burden over the study periods as a consequence of increase in annual funding towards preventive, control and curative interventions. Also, much of this success in the decline could be credited to a combination of sustained economic growth, behavioral change, change in the use of agricultural pesticides or insecticide-like compounds not directly applied for targeting malaria vectors, changes in housing from traditional to modern houses, better surveillance, and improved access to health services in rural areas as a result of newly built health posts reducing the distance from homes to health facilities and an increased number of community health workers [29, 30, 31]. The overall regression predictive model in this study found a strong association between increasing funding towards selected key interventions and reducing occurrence of new cases of of malaria owing to research outcomes indicating about 77% of variations in HMIS-recorded clinical malaria burden could statistically be concurrent to increase in funding towards programs/interventions between the years 2009 and 2018. It is worth noting that, there may be an intriguing link to recent changes in social and ecological sittings that requires further investigation and other potential explanations for the observed decline in disease burden. Hence, need to include an assessment of the potential role of changes in global warming, proper water drainage during rain seasons, and agricultural related use of chemicals, land use, deforestation, and entomological surveillance to reducing the disease burden. Relatedly, the Zambia national malaria program performance review report of 2010, confirms that combined funding for malaria prevention methods (IRS, ITNS, and other vector bone methods) are more compared to treatment and diagnostics methods due to their huge impact on the disease burden [32].

Methodological problems were encountered in this research with primary limitations of this research method being the high cost of research owing to the number of study areas needed in time-series design, the challenges in developing generalizable theoretical principles about community change processes through retrospective data collected, the obscuring of relationships that are unique to a subset of communities, and the problem of diffusion of intervention activities from intervention to control the area of study.

Moreover, it is a cross-sectional study in which the analysis was descriptive and recall bias due to the retrospective nature of data collection related to variables as well as desirability bias may further have an affect the results outcomes of the analysis. In addition, bias-variance may affect the results. Ideally, averaging multiple observations using information from larger regions would reduce the bias—variance from high variance resulting from algorithm modeling the random noise in the data in a model that is over fitted. Also, we conducted our research from the perspective of malaria disease burden routinely recorded in the HMIS-based clinical records while there is also the wider population-level burden seen through population-based household surveys e.g. malaria parasite infection prevalence in DHS, MICS or Malaria indicator survey which typically does not fully access records. In addition, investment will have a lag before the service reaches its beneficiaries. However, an assumption of a one-year time lag was suggested across all interventions while specific interventions such as ITNs distributions leads to protection over 2–3 years, IRS impact limited between 6 and 12 months following spraying and case management benefits in terms of avoiding hospitalization are relatively immediate. Hence, using fixed effects potentially produce similar results and differing on predictor power.

## Conclusion

Malaria funding support is largely targeted towards mass drug administration, indoor residue spray, insecticide treated bed nets, clinical case management (provision of anti-malarial drugs, laboratory diagnostic equipment), entomological intervention, monitoring and evaluation, and information Education/ communication. Further studies are needed to evaluate the relationship between all know malaria interventions against disease burden. Also, finding more efficient and cost-effective ways of developing and evaluating interventions which could be of great benefit to individual health and well-being of a nation or region as addition lives will be saved if we learn how to develop area study interventions more efficiently.

## Supplementary Information

**Additional file 1**. Sources of Malaria Disbursement in Zambia.

**Additional file 2**. Proportion of disbursement towards interventions.

**Additional file 3**. Support towards malaria intervention.

## Data Availability

The dataset used/analysed are available from the corresponding author on request.
