# Supplementary material for: Tracking malaria health disbursements by source in Zambia, 2009–2018: an economic modelling study
Source: Cost Eff Resour Alloc. 2022 Jul 21;20:34. doi: 10.1186/s12962-022-00371-2 (PMC9306103; doi:10.1186/s12962-022-00371-2)
Supplement: Supplementary file 1 — Additional file 1. Sources of Malaria Disbursement in Zambia. [file 12962_2022_371_MOESM1_ESM.docx]

**Additional file 1: Sources of Malaria Disbursement in Zambia**

| Sources of malaria funding/support | Main interventions |
| --- | --- |
| [Africa indoor residual spraying project (AIRS)](http://www.africairs.net/) | IRS |
| [Afrivet](http://www.afrivet.co.za/) | ITNs |
| [Akros](http://akros.com/) | IRS, ES |
| [Bill & melinda gates foundation](http://www.gatesfoundation.org/) | IRS, ITNS, CASE management |
| [Churches health association of zambia (CHAZ)](http://www.chaz.org.zm/) | IRS, ITNS, CASE MANAGEMENT, IEC |
| [Catholic medical mission board](https://cmmb.org/) | IEC |
| [Elimination 8](https://malariaelimination8.org/) | Case MANAGEMENT, ITNS, IRS |
| [End malaria council](https://www.nmec.org.zm/emc) | IEC |
| [First quantum minerals limited](http://www.first-quantum.com/) | IRS, IEC |
| [Global fund to fight aids, tuberculosis and malaria](http://www.theglobalfund.org/EN/) | IRS, ITNS, IEC, M&E |
| [Isdell.flowers cross border malaria initiative](http://www.jcflowersfoundation.org/isdell-flowers-cross-border-malaria-initiative.html) | IEC |
| [Japan international cooperation agency (JICA)](http://www.jica.go.jp/english/) | ITNs |
| [jhpiego](https://www.jhpiego.org/) | IEC |
| [Kagem mine/gemfields](http://corporate.gemfields.co.uk/assets/kagem-emerald-mine-zambia) | IEC, IRS |
| [Konkola copper mines plc (KCM)](http://kcm.co.zm/) | IEC, IRS |
| [Macha research institute](http://malaria.jhsph.edu/malaria-institute-at-macha-miam/index.html) | Case management |
| Malaria control and elimination partnership in africa (MACEPA), a Programme at path | IRS, ITNS, CASE MANAGEMENT, IEC, MDA |
| Malaria modelling consortium (MMC) | ES, MONITORING and evaluation |
| Malariacare, a programme at PATH | Case MANAGEMENT, MDA |
| [Manzi valley](http://www.manzivalley.com/) | Case management |
| Melcome marketing and distributors ltd | ITNS, CASE management |
| Mkushi farmers malaria initiative | IRS, IEC |
| Mopani copper mines | IRS, ITNS, CASE MANAGEMENT, IEC |
| Nakambala sugar | IRS, ITNS, CASE MANAGEMENT, IEC |
| Nwk enterprises | Case management |
| Program for the advancement of malaria outcomes (PAMO) at PATH | MDA, ITNS, CASE MANAGEMENT, MDA |
| [roll back malaria partnership (rbm)](http://www.rbm.who.int/) | case management, ITNS, |
| [Rotarian malaria partners](http://rotarianmalariapartners.org/) | IEC, |
| [Society for family health](http://www.sfh.co.za/) | ITNs |
| [Tableau foundation](https://www.tableau.com/foundation/about) | M&E, IEC |
| [Tropical diseases research centre (tdrc)](http://www.tdrc.org.zm/) | IEC, M&E |
| [Tulane university](https://tulane.edu/) | M&E, CASE management |
| [Unicef](http://www.unicef.org/) | IRS, ITNS, CASE MANAGEMENT, IEC, M&E |
| United states agency for international development (USAID)/president’s malaria initiative (PMI) | IRS, ITNS, CASE MANAGEMENT, IEC, M&E |
| [University of Zambia (UNZA)](http://www.unza.zm/) | IEC, TECHNICAL support |
| [US centers for disease control and prevention (CDC)](https://www.cdc.gov/) | IRS, ITNS, CASE management |
| [US peace corps](https://www.peacecorps.gov/) | case management |
| [Visualize no malaria](http://visualizenomalaria.org/) | IRS, ITNS, CASE MANAGEMENT, IEC |
| [World bank](http://www.worldbank.org/) | IRS, ITNS, CASE MANAGEMENT, IEC, MDA |
| [World health organization (WHO)](http://www.who.int/en/) | IRS, ITNS, CASE MANAGEMENT, IEC |
| [World vision](https://www.worldvision.org/?campaign=119351257&gclid=EAIaIQobChMI6YjCvJPz1AIVBwtpCh1WPApkEAAYASAAEgJbRfD_BwE) | IRS, ITNS, CASE management |
| [Zambia center for applied health research and development (ZCAHRD)](https://ghcorps.org/partners/placement-organizations/zambia-center-for-applied-health-research-and-development/) | ITNs |
| Zambia ministry of community development | M&E, IEC |
| [Zambia ministry of education (MOE)](http://www.moe.gov.zm/) | IEC |
| [Zambia ministry of local government and housing](http://www.mlgh.gov.zm/) | IEC |
| [Zambia national broadcasting corporation (ZNBC)](http://www.znbc.co.zm/) | IEC |
| Zambia national information service (ZANIS) | IEC |

Note;Entomological studies(ES),Insecticide treated nets(ITNs),Indoor residual spraying(IRS),Information education and communication(IEC),Mass drug administration(MDA),Monitoring and evaluation(M&E).
