# Supplementary material for: Tracking malaria health disbursements by source in Zambia, 2009–2018: an economic modelling study
Source: Cost Eff Resour Alloc. 2022 Jul 21;20:34. doi: 10.1186/s12962-022-00371-2 (PMC9306103; doi:10.1186/s12962-022-00371-2)
Supplement: Supplementary file 2 — Additional file 2. Proportion of disbursement towards interventions. [file 12962_2022_371_MOESM2_ESM.docx]

**Additional file 2. Proportion of disbursement towards interventions**
