# Supplementary material for: Tracking malaria health disbursements by source in Zambia, 2009–2018: an economic modelling study
Source: Cost Eff Resour Alloc. 2022 Jul 21;20:34. doi: 10.1186/s12962-022-00371-2 (PMC9306103; doi:10.1186/s12962-022-00371-2)
Supplement: Supplementary file 3 — Additional file 3. Support towards malaria intervention. [file 12962_2022_371_MOESM3_ESM.docx]

**Additional file 3: Support towards malaria intervention**

|  | Intervention | Institutions(count) | Percentage |
| --- | --- | --- | --- |
| 1 | Information education & communication | 26 | 24.1 |
| 2 | Case management | 22 | 20.4 |
| 3 | Indoor residue spray | 23 | 21.3 |
| 4 | Insecticide treated nets | 21 | 19.4 |
| 5 | Monitoring & evaluation | 8 | 7.4 |
| 6 | Mass drug administration | 5 | 4.6 |
| 7 | Entomological studies | 3 | 2.8 |
|  | **Total interventions** | **108** | **100** |
